# Prevalence of chronic non-communicable diseases in Ethiopia: A systematic review and meta-analysis of evidence

**DOI:** 10.3389/fpubh.2022.936482

**Published:** 2022-08-03

**Authors:** Fisaha Haile Tesfay, Christina Zorbas, Laura Alston, Kathryn Backholer, Steven J. Bowe, Catherine M. Bennett

**Affiliations:** ^1^Institute for Health Transformation, Deakin University, Geelong, VIC, Australia; ^2^College of Medicine and Health Sciences, School of Public Health, Mekelle University, Mekelle, Ethiopia; ^3^Deakin Biostatistics Unit, Faculty of Health, Deakin University, Geelong, VIC, Australia

**Keywords:** NCD, non-communicable diseases, Ethiopia, systematic review, cardiovascular diseases

## Abstract

**Background:**

Non-communicable diseases (NCDs) are a growing global health challenge disproportionately impacting low- and middle-income settings, including Ethiopia. Currently, the body of evidence describing the burden of NCDs is fragmented, inconsistent, health facility- or institution-based, and out-dated in Ethiopia. We conducted a systematic review of the literature and meta-analysis of the prevalence of NCDs in community settings in Ethiopia.

**Review methodology:**

Community-based quantitative studies published in English between January 1st, 2012, and June 30th, 2022, that reported on the prevalence of NCDs in Ethiopia were included. A systematic search of Medline, Embase, Scopus, CINAHL, and Global Health using pretested search terms related to NCDs was conducted, and data were extracted using a piloted data extraction proforma adapted from the Joanna Briggs Institute tool. Meta-analysis was performed using Stata 16. While the pooled prevalence of Diabetes Mellitus (DM) and undiagnosed (DM) was computed and presented using forest plots, then overall prevalence of NCDs and other various types of NCDs were narratively synthesized. *I*^2^ was used to assess heterogeneity. Studies that did not fulfill the criteria (used similar tool to measure the types of NCDs) for meta-analysis were narratively synthesized.

**Results:**

Twenty-two studies met the inclusion criteria. Five studies measured the prevalence of NCDs (all NCDs together), ranging from 29 to 35% (prevalence estimates not pooled). The pooled prevalence of Diabetes Mellitus (DM) across ten studies was 5% (95% CI: 4–7%). Three studies each reported on the prevalence of undiagnosed DM (pooled prevalence 5%, 95% CI: 4–7%) and pre-DM (pooled prevalence 7%, 95% CI: 3–14%%). In a narrative analysis the prevalence of cardiovascular conditions ranged from 13.4 to 32.2% (*n* = 3 studies), cancer mortality ranged from 4 to 18% (*n* = 3 studies) and respiratory conditions ranged from 1 to 18% (*n* = 3 studies). Some studies have determined more than one NCDs and that is why the total number of studies are exceeding more than twenty studies.

**Conclusion and recommendations:**

Our analysis found that approximately one-third of Ethiopians have an NCD, with cardiovascular diseases the most common of all NCDs. The prevalence of respiratory conditions also appears high, but there are insufficient data for a pooled estimate. Whilst the prevalence of DM appears relatively low, there is evidence that the magnitude is increasing. Public health actions to address the high burden of cardiovascular and respiratory diseases, as well as the increasing magnitude of DM in Ethiopia, must be prioritized.

**Systematic review registration:**

PROSPERO [CRD42020196815].

## Introduction

Non-communicable diseases (NCDs) are a growing international health challenge. NCDs account for 71% of the total global deaths (1) and more than half of the global burden of diseases (2). According to the 2019 World Health Organization report on World Health Statistics, the negative impact of NCDs is highest for low and middle-income countries (LMICs) (3). NCDs account for 78% of all deaths, 85% of premature deaths, and 50% of disabilities in LMICs (1, 4). Cardiovascular diseases (CVDs) contribute to the majority of NCD-related mortalities in LMICs (2), followed by cancers, respiratory diseases, and Diabetes Mellitus (DM) (5). Like most low-income countries, NCDs were the leading cause of age standardized death rate in causing 711 deaths per year/100,000 people (95% Uncertainty Interval: 468.8–1,036.2) in Ethiopia (6).

Between 1990 and 2015, life expectancy in Ethiopia increased by 18 years (7) alongside a reduction in communicable diseases, maternal and neonatal mortality, and nutritional deficiencies (8). In contrast, some evidence suggests that the magnitude of NCDs increased in Ethiopia during this time (9, 10). Various factors such as epidemiologic, demographic, socioeconomic and nutrition transitions have contributed to this growing trend (11).

Despite the emerging concern about NCDs in Ethiopia, there is no national reporting of NCDs. To our knowledge there has only been one previous review conducted from 1990 to 2011 to describe the prevalence of NCDs in Ethiopia, and the key findings include: the prevalence of cardiovascular diseases (CVDs) ranged from 7.2 to 24%, cancer prevalence was 0.3%, DM ranged from 0.5 to 1.2%, and asthma ranged from 1 and 3.5% (12). However, it has been almost 10 years since this review was conducted and, given the social and economic transitions that Ethiopia is experiencing, we expect a substantial change in the prevalence of NCDs in the intervening years. Furthermore, the studies included were mostly focused on NCDs captured in health facility-settings. So selection bias may have contributed to the overestimation of the prevalence of NCDs due to over selection, or underestimation due to ascertainment of more severe presentations of these conditions (13). Community based studies have been shown to represent a more accurate population prevalence of disease (14). For instance while the prevalence of DM was 1.9% in a community based study (14), the prevalence of DM in a study conducted in a hospital with similar catchment population to the former study was 12.2% (15).

In addition, recent evidence describing the prevalence of NCD morbidity and mortality in Ethiopia is fragmented and inconsistent, with some reporting a relatively higher prevalence of NCDs (34.5%) (10), whilst others reported this to be very low at 1.7% (16). Hence, we aimed to conduct an updated systematic review of the literature and meta-analysis of the population prevalence of NCDs in Ethiopia from community-based studies.

## Methods

### Protocol

A protocol for this systematic review and meta-analysis was registered in PROSPERO (registration number is CRD42020196815). This review was guided by the Preferred Reporting Items for Systematic Reviews and Meta-Analyses (PRISMA) (17).

### Context/setting

Ethiopia is located in the Horn of Africa and it is the second-most populous country in sub-Saharan Africa after Nigeria with a total population of more than 99,01 million (as of July 2021) and a population growth rate of 2.6% per year (18). According to the 1994 constitution, Ethiopia is administratively structured into nine regional states: Tigray, Afar, Amhara, Oromiya, Somali, Benishangul-Gumuz, Southern Nations Nationalities and Peoples (SNNP), Gambela, Harari and Sidama; and two city council administrations which include Addis Ababa and Dire Dawa (19). In 2018, life expectancy in Ethiopia was 63.2 years (20). The major health problems in Ethiopia include HIV and AIDS, tuberculosis, malaria and nutritional deficiencies, maternal and child health along with growing concern for non-communicable diseases such as cardiovascular disease, diabetes mellitus, chronic respiratory disease and injuries (21, 22). While Tigray, Amhara, Oromia and SNNP are considered to be relatively developed regions, the remaining are less developed and perhaps the population that lives there are mostly nomads.

### Searches

Studies accessible *via* Medline, Embase, Scopus, CINAHL, Global Health and published between January 1st, 2012 and June 30th, 2022 were searched and reviewed. The timeframe was selected to update a similar systematic review on the topic summarizing literature up to the end of 2011 (12). The search terms were prepared based on an initial scoping review using terms related to epidemiologic parameters AND “Non-communicable disease” AND “Ethiopia”. These search terms were combined to inform a systematic search strategy that was applied across all five academic databases (Table 1). The initial search was part of a broader NCD epidemiology project, but only those studies reporting NCD prevalence are included in this review. In addition, the first 150 hits from Google Scholar, gray literature databases such as ProQuest, open gray, government websites (Ethiopian Ministry of Health and Ethiopian Public Health Institute), NGO websites such as the WHO, and the reference lists of selected papers were also searched for additional relevant literature.

**Table 1:** Systematic search strategy applied across five academic databases from 2012 to July 2020 (e.g., this is the Medline search).

**Hedge 1: indicators**	**Hedge 2: non-communicable disease**	**Hedge 3: Ethiopia**
Prevalence*OR Proportion* OR Magnitude* OR Epidemiology* OR Pattern*OR Trend* OR Burden	Non-communicable diseases*, chronic disease* or chroni illness* or chronic disease [MeSH Terms] *, non-communicable disease*, NCD* OR Non-communicable disease [MeSH Terms] **OR** Cardiovascular disease*, OR (cardiovascular diseases [MeSH Terms]) CVD*, heart disease* OR stroke* OR Myocardial infarction* OR Diabet* OR diabetes mellitus [MeSH Terms]), DM **OR** (cancer) OR neoplasms [MeSH Terms]) or neoplasm* or Sarcoma **OR** (Chronic obstructive pulmonary disease) OR chronic obstructive pulmonary disease [MeSH Terms]) OR copd[MeSH Terms]) OR copd) OR lung disease) OR lung disease[MeSH Terms])	Ethiopia OR Tigray OR Amhara PR Oromia OR Afar OR Somali OR Gambela OR Benshangul Gumuz OR Addis Ababa OR Diredawa OR

### Study selection

Primary quantitative publications reporting on the magnitude of NCDs (morbidity or mortality) as a pooled (more than one type of NCD combined) or individual NCD types (e.g., diabetes) were included. We only included community-based cross-sectional studies and baseline prevalence estimates reported from longitudinal studies. The reason we include only community-based studies is because institution-based studies are prone to selection bias (e.g., may underestimate prevalence because they may not capture more serious diseases). We also excluded studies with no clear objective, research question, or methodologies, and studies with very small sample sizes (*n* <50) because of the poor reliability of the estimates.

All search results were exported to Endnote and then to Covidence, and duplicates removed, records screened by title and abstract, and full texts screened for those that remained eligible after initial scans. Fisaha Tesfay (FT) (100%), Laura Alston (LA), and Christina Zorbas (CZ) (50% each) conducted screening by title and abstracts for relevance, then all retained articles underwent full-text screening against the inclusion and exclusion criteria by two reviewers, independently. A third reviewer was consulted in circumstances of disagreement between the two reviewers and consensus was reached. All reasons for the exclusion of a paper were documented.

### Data extraction

Data were extracted using a pretested data extraction proforma. It was adapted from the Joanna Briggs Institute (JBI) template (23) and informed by the literature on NCD prevalence to ensure the relevance and uniformity of the extracted data. The data extraction proforma was piloted by two independent reviewers on three purposively selected papers based on their study design (cross sectional) and type of NCD prevalence reported (DM and total NCDs combined). The data extraction included author and year, study aims, study design, study population, sample size and sampling technique, data collection and analysis methodologies, outcomes, the prevalence of NCDs, demographic characteristics (age, gender, residence (urban/rural) educational status, employment), authors' conclusions, and limitations of the study. All prevalence estimates of NCDs, including those provided for study sub-groups estimates were also extracted. The outcome variables in this review included overall/subgroup prevalence (%) of NCDs and prevalence of NCDs in Ethiopia by type of NCD condition, e.g., CVD, DM, cancer, and respiratory diseases. LA and CZ each extracted data from a subset of the studies (10%) and cross-checked for accuracy while the lead author (FT) extracted 100% of the data.

### Data analysis and synthesis

Studies measuring the prevalence of NCDs were summarized overall and according to NCD type. Sub-group analyses included stratification of analysis by region in Ethiopia. Prevalence estimates were pooled using a random-effects meta-analysis using the **metaprop** command in Stata. The **metaprop** command is used to combine proportions using the binomial distribution to model the within-study variabilities or Freeman-Tukey double arcsine transformation to stabilize the variances (24, 25). Pooled prevalence estimates are presented visually using forest plots. Assessment of heterogeneity was possible for Diabetes Mellitus given the number of studies were 10 but heterogeneity for undiagnosed Diabetes was not possible to assess because the number of included studies were less 10. The *I*^2^ for the pooled prevalence of diabetes was 94.49 with *p* < 0.001 (Figure 2). To address this, we conducted sub-group analysis by regional states (Figure 2) but the heterogeneity for the sub-group analysis is also high because the included studies in the sub-groups (regional states) were less 10.

### Assessment of methodological quality

The Newcastle-Ottawa scale for critical appraisal of cross-sectional study designs was used to assess the quality of the included studies and the scores for each study were determined based on three criteria which involve selection (five stars maximum), comparability (two stars maximum), and outcome (three stars maximum) (26). Under these three broad parameters, there are eight quality indicators to assess a study. The Newcastle-Ottawa Scale does not recommend a cut-off point between high, moderate, and low-quality studies. Hence, a relative comparison of the studies was made.

FT assessed the quality of all included studies, with LA and CZ each appraising 10% of these studies, with minor discrepancies observed and resolved through discussion. For all included studies, any quality assessment uncertainties were discussed between FT, LA and CZ. Quality scores were not used to exclude studies, but rather to identify any limitations in the body of evidence being summarized or included in the meta-analysis.

Study quality scores ranged from four to ten stars. Out of the 22 studies, 11 studies scored ten out of ten (10, 16, 27–35), four studies scored nine out of ten (9, 36–38), two studies scored eight out of ten (14, 39), two studies scored seven out of ten (40, 41), two studies scored six out of ten (42, 43) and one scored four out of ten (44). The major quality issues for the study that scored four out of 10 included not reporting non-response, not describing data collection tools, not controlling for confounding, inadequate statistical testing, and unjustified small sample sizes. The major quality issues also reflect the limitation seen across other lower scoring studies.

## Results

### Study characteristics

**Figure 1:**
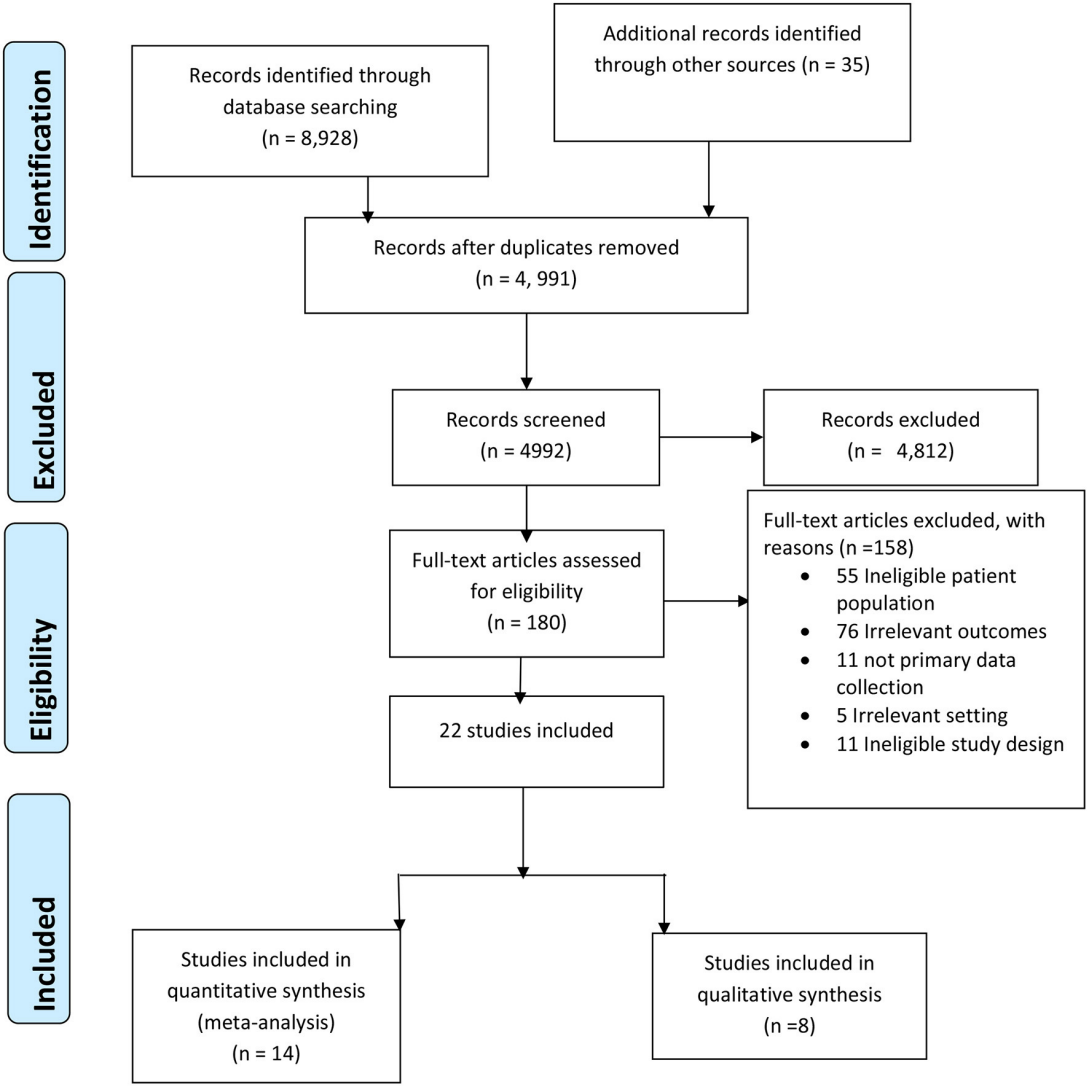
PRISMA 2009 flow diagram.

After removing duplicates, 4,991 papers were screened by title and abstract, and 180 papers fulfilled the criteria for full-text screening (see Figure 1). The full-text review resulted in 13 papers that were included in meta-analysis and these studies were related to diabetes mellitus (DM) and undiagnosed DM. Ten studies estimated the prevalence of DM and three studies reported on undiagnosed DM. One study that measured DM, also measured mortality due to CVD, respiratory conditions and cancer (9).

Eight studies including two gray literature were narratively synthesized. Three studies reported on the magnitude of NCD morbidities and mortalities (total), CVDs, cancer and respiratory conditions (10, 16). Generally, while proportion of CVDs and cancers, proportion of respiratory conditions were reported in three studies, NCDs (all NCDs combined) were reported in five studies.

Major reasons for excluding of studies were the use of an ineligible population (e.g., prevalence among children) or irrelevant outcomes (e.g., some studies compared measures of adiposity and others measured breast cancer pathology) where none reported the prevalence of NCDs.

Twenty studies used a cross-sectional study design, and two studies reported baseline data from longitudinal study designs.

Three studies reported on comorbidities. The first study reported that among individuals with DM, the prevalence of hypertension was 61.1% (29) while the second study reported that 3.6% of study participants had two cases of NCDs per household (16). The third study reported that the number of NCD identified per person is ranged from one to four (39). The commonest comorbidities included hypertension (63.5%), diabetes (42.5%) and heart disease (25.6%) (39).

The sample size of the included studies varied depending on the NCDs examined and sources of data, ranging from 385 to 67,397.

### Prevalence of non-communicable disease

Meta-analysis was conducted for DM prevalence (diagnosed and undiagnosed) while the results for the overall NCD prevalence, CVDs, cancers and respiratory diseases all narratively synthesized due to the heterogeneity of the estimates.

### NCDs combined

While two studies reported on the magnitude of mortality due NCDs (9, 10), a further three studies reported on the prevalence of NCDs (16, 34, 41). The mortality studies from 2009 to April 2015 and 2009 to 2011 that were conducted in Tigray-(northern Ethiopia) from a population-based study reported that the NCD-related mortality rates were 34.5 and 28.6%, respectively. These two studies were conducted in a research surveillance center in Tigray (Supplementary Table 1). Morbidity as reflected in the prevalence of NCDs was reported in a separate study in a similar research setting in northwestern Ethiopia (Amhara regional state) reported that the prevalence of NCDs in 2014 was as low as 1.2% (16). A study by Muluneh et al. (44) conducted in southwestern Ethiopia (Oromia regional state) in a field research center [Gilgel Gibe Field Research Center (GGFRC)] reported that the prevalence of NCDs from 2008 to January 2009 was 8.9% (41). Furthermore, another study conducted among long truck drivers (from Djibouti port to Ethiopia) on the prevalence of NCDs found that 28.5% of them have NCDs (34). In a factsheet prepared by the WHO for the year 2018, NCDs were reported to contribute to 39% of all deaths (45). The source of data for the WHO fact sheet are civil registration and vital statistics systems, household and other population-based surveys, routine health-facility reporting systems and health-facility surveys, administrative data systems and surveillance systems.

### Prevalence of diabetes mellitus

**Figure 2:**
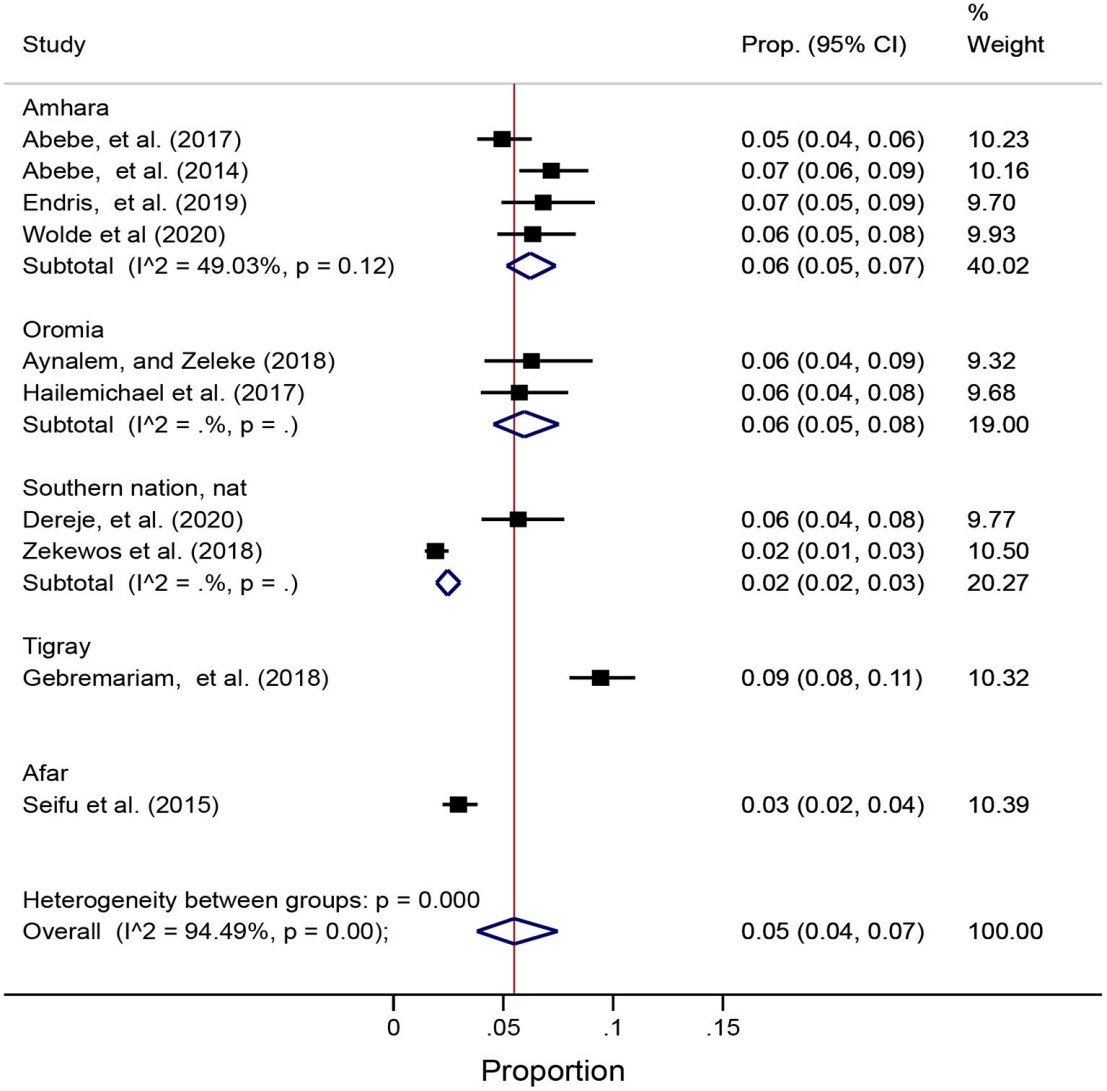
Forest plot of pooled prevalence of diabetes mellitus overall and by regional states in Ethiopia using the random effect model.

Ten studies reported on the prevalence of DM (Supplementary Table 1). Regional distribution of these studies included four studies from Amhara (16, 27, 30), two studies from Oromia (28, 44), one study from Tigray (43), two studies from Southern Nation, Nationalities and Peoples' regional states (14, 29) and one study from Afar (42). Using the random effect model, the pooled prevalence of DM was 5% with 95% CI (4–7%). In a sub-group analysis, the pooled regional distribution of DM ranged from 2% in the Afar regional state to 6% in Amhara and Oromia regional states (Figure 2).

*I*^2^ for the pooled prevalence of Diabetes was 94.49 with *p* < 0.001 (Figure 2). To address this, we conducted sub-group analyses by regional states (Figure 2) but the heterogeneity between the regions in Ethiopia remained high, likely due to differences in regional states because the number of studies were fewer than 10 studies.

One study conducted among long distance truck drivers at national level in Ethiopia reported that the prevalence of DM was 8% (34). Another study conducted in Tigray the Tigray region found that diabetes mellitus contributed to 1.7% of deaths (9).

### Undiagnosed DM

**Figure 3:**
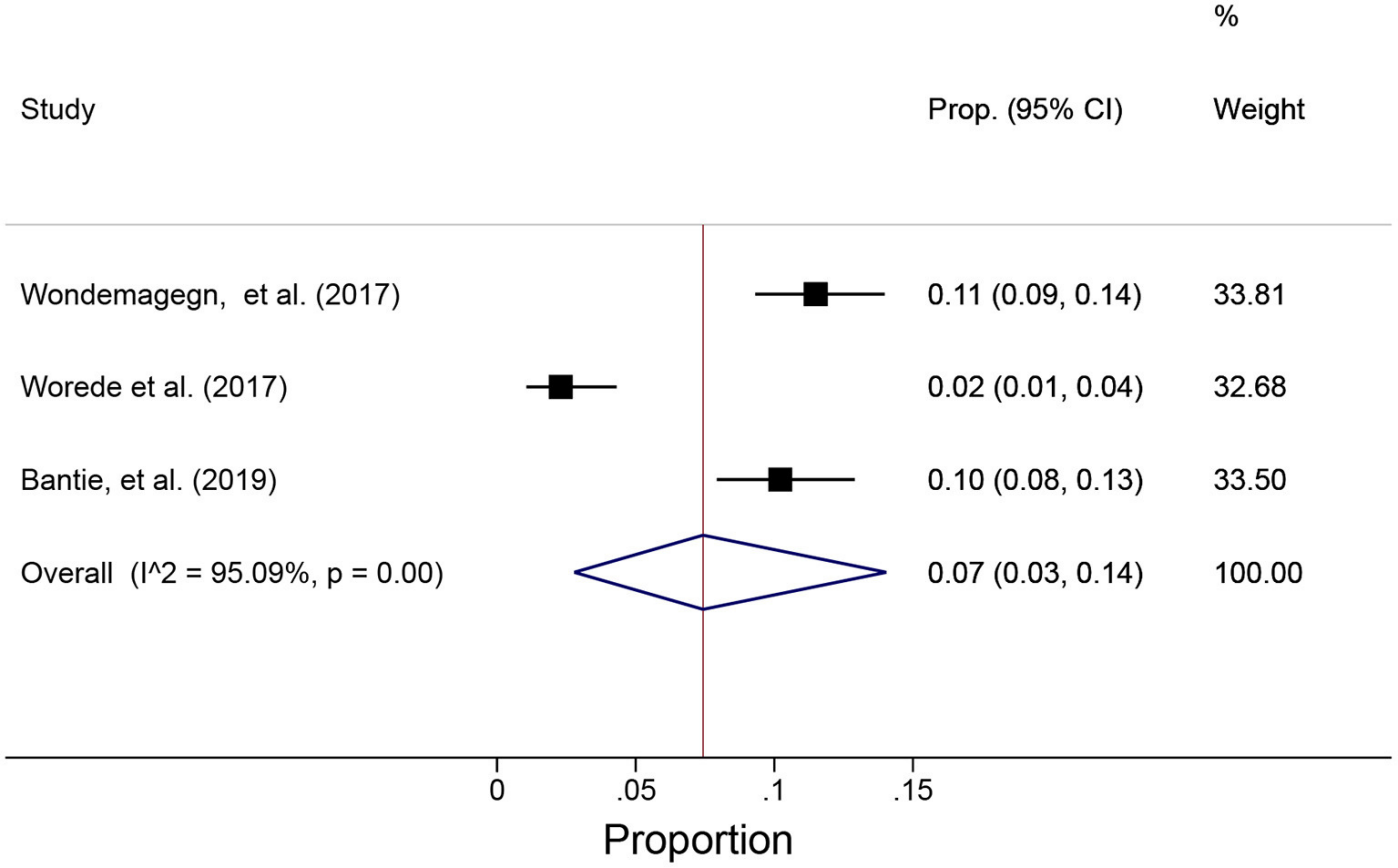
Shows the pooled prevalence of undiagnosed DM using the random effect model.

Three studies conducted between 2017 and 2019 estimated the prevalence of undiagnosed DM and ranged from 2.3 to 11.5% (Supplementary Table 1) (31, 32, 36). All of these studies were conducted in Amhara regional state of Ethiopia. In the first study, the prevalence of undiagnosed DM was 11.5% (31). This study was conducted among people aged >25 years. Nearly 61% of study participants were living in rural areas and 59% of them were males (31). Similarly, the prevalence of undiagnosed DM in the second study was 2.3% (32). This study was conducted in a rural town among people age >20 years and majority (56%) of the study participants were females (32). In the third study, the prevalence of undiagnosed DM was 10.2% (36). This study was conducted among people aged >18 years of and the study and the mean age of the participants was 35.2 (±13.8) years; about 13.5% of participants had no formal education (36). The pooled prevalence of the undiagnosed DM was also estimated using the random effect model and the overall prevalence was 7%, 95% CI (3–14%) (Figure 3).

Between studies heterogeneity was assessed but due to the small number of studies, it remained high (*I*^2^ 95.1 with *p* ≤ 0.001) (Figure 3).

### Cardiovascular diseases

Three studies reported on the prevalence of CVD morbidity or mortality (9, 10, 16) (Supplementary Table 1). According to Abebe et al., the prevalence of CVD morbidity was 32.2% in 2014 (16). This study was conducted in a demographic surveillance site in northwestern Ethiopia (Amhara regional state) among a sample of 67, 397 individuals. another demographic surveillance system (Dabat Health and Demographic Surveillance System). Half of the study participants were women and 75.1% lived in rural area (16). Weldearegawi and his colleagues analyzed the cause of mortality among 409 deceased individuals using verbal autopsy and found that 13.4% of deaths were due to circulatory system diseases in the Tigray regional state (9). This study was conducted in demographic surveillance site (Kilte Awlealo demographic and surveillance site) –where demographic and social changes of a geographically defined population is monitored. The total population in the surveillance site involved 68, 495 (51.4% are women). Among the causes of death related to the circulatory system diseases, 7.3% were due to cerebrovascular diseases, 2.7% were due to ischemic heart disease, 2.2% were due to congestive heart failure and 1.2% were due to other CVDs (Rheumatic heart diseases and hypertension) (9). In another study by Abera et al. in northern Ethiopia, who also examined the cause of death using verbal autopsy, 41.8% of deaths were found to be due to cardiovascular causes (from 1,091 deceased individuals between 2009 and 2015) (10). This study was conducted in a similar setting and geographic location to the study by Weldearegawi et al.

### Cancers

Three studies, conducted between 2009 and 2011, reported on the prevalence of cancer morbidity and mortality (9, 10, 16) (Supplementary Table 1). Abebe et al. reported that the prevalence of cancer in 2014 was 3.2% (16). The second study by Abera et al., which used verbal autopsy to determine the cause of death, reported that from 2009 to April 2015, 18.1% of deaths were due to cancer (10). Similarly, the third study by Weldearegawi et al. used verbal autopsy to determine that 4.4% of deaths were due to cancer from 2009 to 2011 (9). While the studies by Weldearegawi et al. and Abera et al. are conducted in northern Ethiopia (Tigray region) as stated above in the cardiovascular diseases section, the study by Abebe et al. (16) was conducted in northwest Ethiopia (Amhara regional state).

### Respiratory diseases

Three studies reported on the prevalence of mortality and morbidity from respiratory diseases (9, 10, 16) (Supplementary Table 1). One longitudinal study which was conducted from 2009 to 2015 examined the magnitude of NCDs in a demographic and health surveillance site in Northern Ethiopia (10) and reported that the prevalence of chronic obstructive disease (COPD) was 17.8% in 2009. Another longitudinal study that was conducted from 2009 to 2011 examined the causes of death using physician assigned verbal autopsy. This study found that 1.2% of deaths were attributed to respiratory disease causes (9). In another study by Abebe et al. (reported above), conducted in northwestern Ethiopia (Amhara regional state), the prevalence of Asthma was 27.7% in 2014 (16).

## Discussion

This systematic review and meta-analysis provides an up-to-date synthesis of the NCD prevalence literature (in relation to DM, CVDs, cancer and respiratory disease) in Ethiopia. Meta-analysis was done only for DM and undiagnosed DM. Heterogeneity remained to be high for the two studies due to the small number of studies included in meta-analysis. Hence, we recommend further meta-analysis studies that involve more studies to allow estimate with more precise comparability within the regional states in Ethiopia. We found prevalence studies of NCDs to be scant, inconsistent, making it impossible to report precise estimates by region. To adequately monitor and address NCDs in Ethiopia, a routine population-level data collection system should be established. Doing so will support population-level policies and targeted interventions to high risk groups to reduce the burden of NCDs in Ethiopia.

With NCD mortality (as a proportion of all deaths) ranging from 28.6% (9) to 34.4% (10), findings in the current review are similar to the proportion of deaths that can be attributed to communicable disease deaths (33%) in Tigray regional state (10). This is consistent with the findings of a study that was conducted in African and Asia which reported that NCD caused up to 35.6% mortality (46).

On the other hand, the prevalence of NCD morbidity was notably lower in northwestern Ethiopia (Amhara regional state) which was 1.2% (16). However, in a study that was conducted in sub-Saharan Africa, NCDs contributed to 67% of disability adjusted life years (8). The considerably lower magnitude of NCD morbidity in the study by Abebe et al. in Ethiopia was likely related to methodological shortcomings. Use of self-report to ascertain NCD outcomes is likely to have led to the underestimation of the prevalence of NCDs (16). Whilst our results on the prevalence of NCDs in Ethiopia are not directly comparable to a similar 2012 review (21), it appears that there is no conclusive evidence to compare the overall trend of NCDs mortality and morbidity in the last 10 years. The review that was done 10 years ago by Misganaw et al., did not provide any evidence regarding mortality and morbidity due to NCDs (total) (21).

Global evidence suggests that the prevalence of DM is growing (47, 48) including in Ethiopia (49). The prevalence of DM in a previous (2012) systematic review in Ethiopia ranged from 5 to 5.3% in a community based studies (21). The pooled prevalence of DM in our meta-analysis was 6% with considerable differences between regional states. In the current review, the highest prevalence was reported in Amhara from 2014 to 2019 and Oromia regional states from 2017 to 2018, and the lowest was in Afar regional state in 2015. The higher prevalence of DM estimated in our study compared to prior studies, is supported by studies across other countries in sub-Saharan Africa. For example, a systematic review from Nigeria found that DM increased from 2.0% in 1990 to 5.7% in 2015 (50). Other studies from other parts of sub-Saharan Africa have found a comparable magnitude of DM (51–55).

Globally in 2017, sub-Saharan Africa had the highest proportion of undiagnosed DM (66.7%) (48). Among those with DM, 70% of them were unaware of their DM condition (56). The findings of the current meta-analysis showed that the prevalence of undiagnosed DM was slightly higher (7%) than other studies from sub-Saharan Africa (57, 58). For instance, the prevalence of undiagnosed-DM in one systematic review from 2007 to 2020 among adults in African countries was 3.85% (57) while another systematic review reported that the prevalence was 5.37% (58). Similar to the findings of our study, the prevalence of undiagnosed DM in Nigeria has been estimated at 7.8% in a systematic review conducted from 1990 through 2018 (50).

Global evidence suggests that cardiovascular diseases (CVDs) contributed to one-third of global deaths in 2015 (59). The proportion of all deaths that can be attributed to CVDs ranged from 13.4% (9) to 41.8% (10) in our review. Our finding is similar to the 16% of CVD attributed deaths in Ethiopia reported by the WHO (45). Furthermore, according to a systematic review conducted in sub-Saharan Africa from 1990 to 2019, CVDs were responsible for ~13% of all deaths and 37% of all NCD-related deaths (60). In another study from northwestern Ethiopia (Amhara), 32.2% of all deaths were attributed to CVD (16). The high proportion of CVD deaths is not unique to Ethiopia; increasing CVD morbidity and mortality has been reported across sub-Saharan Africa (61, 62).

Two factors may be contributing to the increasing prevalence of CVDs. These factors include sociodemographic factors and a shift from a more agrarian lifestyle to industrial and service-related employment. For instance, evidence shows that, compared with non-agricultural workers mainly living in urban areas, rural agriculture workers have a lower prevalence of hypertension, overweight and obesity; and a higher prevalence of underweight and smoking in low and middle income countries (63). Overall, the findings of the current review align with existing evidence that supports the presence of an epidemiological transition in Africa over the last decade (64).

In 2017, there are 24.5 million cancer cases globally and 9.6 million cancer deaths (65). The burden of cancer is increasing in low and middle income countries (66) and Africa contributes ~6% of global cancer cases (67). Epidemiological studies of the prevalence of cancer are scarce in Ethiopia with only two studies included in this systematic review. The first study reported the prevalence of cancer morbidity to be 3.2% (16) while in second study the attributable fraction of cancer mortality was 18.1% of all NCD-related deaths (10). Additional studies describing the burden of cancer in Ethiopia are urgently required.

Globally, chronic obstructive pulmonary diseases (COPD), contributes to 3.9 million deaths and affects 5% of the global population (68). In our systematic review, one study reported on the prevalence of COPD in a single study, which was 17.8% in 2014 (16) and one further study which was conducted from 2009 to 2011 reported that 1.2% of NCD-related deaths were from respiratory causes (9). This finding is similar to the 2018 WHO report where COPD contributed to 2% of all NCD deaths (45). While the burden reported in the current review is larger than the global, it is within range for the prevalence of COPD in sub-Saharan Africa; 4.1% to almost 22.2% (68). The relatively low prevalence in Ethiopia compared to many other sub-Saharan African countries may be due to the difference in the country level factors, particularly the stage of epidemiological transition, and measurements used (e.g., diagnostic techniques) (68, 69).

This systematic review and meta-analyses is not without limitations. Except for DM, a meta-analysis was not possible for other NCDs due to the high heterogeneity between the remaining studies. As with all reviews, this systematic review and meta-analysis is limited by the findings and quality of the individual studies. For instance, some studies used defined age groups by a minimum age (e.g., > 40) to determine the prevalence of DM which could potentially overestimate the magnitude. Furthermore, in studies that used verbal autopsy, a notable proportion of deaths were recorded as either unspecified (16.8%) (10) or undetermined (3.6%) (9). This potentially underestimate the magnitude of NCDs.

Key risk factors for NCDs in Ethiopia include family history, hypertension, tobacco smoking, and a high body mass index and prior NCD prevalence and co-morbidity (51–53). Urbanization and income status enhance these risk factors (47, 70). For instance, urbanization is associated with overweight because of limited physical activity and increased access to unhealthy foods in an urban environment (70). In addition, existing evidence suggests that the most common risk factors for COPD include biomass combustion and cigarette smoking (71, 72). Future research is required to systematically summarize the prevalence of these risk factors to better inform future health and policy priority setting in Ethiopia.

In conclusion, in the current review of the limited evidence available, CVDs are identified as the most common NCDs in Ethiopia. The prevalence of NCDs appears to be high and increasing in Ethiopia, but comprehensive, regular and reliable surveillance data is lacking. Given the heterogeneous nature of the available prevalence studies and the epidemiological transition that is taking place, it is essential that the establishment of a national NCD mortality and morbidity surveillance system is prioritized and implemented in Ethiopia. This will be essential to inform policy and practice to reduce the current and future burden of NCDs in Ethiopia.

## Data availability statement

The original contributions presented in the study are included in the article/Supplementary material, further inquiries can be directed to the corresponding author/s.

## Author contributions

FT conceptualized, designed the study, conducted the search, analyzed, and interpreted the data as well as drafted the manuscript. KB and CB conceptualized, designed, critically reviewed the design, analysis and interpretation of the study, and critically reviewed and approved the manuscript. CZ and LA critically reviewed the design and analysis of the study and critically reviewed and approved the manuscript. SB analyzed data, critically reviewed, and analyzed and approved the manuscript. All authors contributed to the article and approved the submitted version.

## Funding

This study is part of a postdoctoral fellowship and Deakin University has funded the fellowship of FT (paying salary). Deakin University has no involvement in the design, collection, analysis, interpretation and in writing of the manuscript.

## Conflict of interest

The authors declare that the research was conducted in the absence of any commercial or financial relationships that could be construed as a potential conflict of interest.

## Publisher's note

All claims expressed in this article are solely those of the authors and do not necessarily represent those of their affiliated organizations, or those of the publisher, the editors and the reviewers. Any product that may be evaluated in this article, or claim that may be made by its manufacturer, is not guaranteed or endorsed by the publisher.
