# Supplementary material for: Prevalence of chronic non-communicable diseases in Ethiopia: A systematic review and meta-analysis of evidence
Source: Front Public Health. 2022 Aug 3;10:936482. doi: 10.3389/fpubh.2022.936482 (PMC9385028; doi:10.3389/fpubh.2022.936482)
Supplement: Supplementary file 1 [file Table_1.DOCX]

|  | | **Table 1:** Shows the characteristics and quality scores of included studies | | | | | |
| --- | --- | --- | --- | --- | --- | --- | --- |
| **Authors name and year** | **Region** | | **Sample size** | **Measurement of outcomes** | **Measured outcome** | **Prevalence (%)** | **Quality Score** |
| Abebe, et al (2017) | Amhara | | 67 397 | Structured interview administered questionnaire on various NCDs was used. | Diabetes | 4.90% | 10 |
|  |  |  |  |  | NCDs combined | 1.2% |  |
|  |  |  |  |  | Cardiovascular diseases | 32.2% |  |
|  |  |  |  |  | Cancer | 3.2% |  |
|  |  |  |  |  | Respiratory diseases | 4.9% |  |
| Abebe, et al. (2014) | Amhara | | 1100 | Face to face interview using the WHO steps survey tool  *peripheral blood samples by figure puncture was taken  *Anthropometric measurements using standardised and calibrated equipment’s was take | Diabetes | 7.2% | 10 |
| Abera, et al. (2017) | Tigray | | 67 397 | *Lay trained data collectors conducted the data collection  *Physician review verbal autopsy to identify the most probable cause of death.  *Principal component analysis was done to estimate socioeconomic index | NCD combined | 34.50% | 10 |
|  |  |  |  |  | Cardiovascular | 41.80% |  |
|  |  |  |  |  | Cancer | 18.1% |  |
| Aynalem et al. (2018) | Oromia | | 414 | Data on demographic and behavioural characteristics was collected  *The questionnaire was adopted from the WHO stepwise approach for noncommunicable disease | Diabetes | 6.5% | 10 |
| Bantie, et al. (2019) | Amhara | | 607 | *The questionnaire involved sociodemographic, behavioural and clinical characteristics and clinical characteristics and was developed from the literature  *Interview administered questionnaire was used to collect the data. | Undiagnosed Diabetes | 10.2% | 9 |
| Dereje, et al. (2020) | (SNNP) | | 634 | Face to face interview was conducted using the WHO steps survey  *Information on tobacco use, alcohol consumption, fruit and vegetable consumption, physical activity, physical measurement, blood glucose level, chronic disease history and family health was collected | Diabetes | 5.7% | 10 |
| Desalegn et al. (2017) | Oromia | | 576 | Data were collected using the World Health Organization standardized structured questionnaire on cardiovascular risk assessment for developing countries | Cardiovascular diseases | 23.8% | 7 |
|  |  |  |  |  | Diabetes | 6.2% |  |
| Endris, et al. (2019) | Amhara | | 587 | *Sociodemographic and behavioural characteristic and biochemical data were collected  *The WHO steps survey questionnaire was used. | Diabetes | 6.8% | 10 |
| Eyowas et.al (2022) | Amhara | | 1440 | Interview and review of medical records | Comorbidities to NCDs | 1.4% | 8 |
| Gebremariam, et al. (2018) | Tigray | | 1,527 | *The WHO modified steps survey tool was used to collect data | Diabetes | 9.40% | 6 |
| Hailemichael et al. (2017). | Oromia | | 576 | *Data were collected using the WHO standardized structured questionnaire on cardiovascular risk assessment for developing countries  *CVD risk factor scores were computed by assigning a score of +1 to each independent variable | Diabetes | 6.2% | 4 |
| Muluneh , et al. (2012) | Oromia | | 5,000 | *Interviewer administered questionnaire adopted from the WHO steps survey was used. After the first interview, participants were also given an appointment for a physical measurement | NCD combined | 8.90% | 7 |
| Seifu et al. (2015) | Afar | | 1861 | It was collected using the WHO steps survey. | Diabetes | 3.8% | 6 |
| Vinodhini et al. (2017) | Oromia | | 380 | Data was collected using self-administered questionnaires; anthropometric data and blood biochemistry. | Prevalence of pre-DM | 19.5% | 9 |
| Weldearegawi et al. (2013) | Tigray | | 409 | Data were collected from surveillance site and verbal autopsy was used to collect data. | Diabetes | 1.70% | 9 |
|  |  |  |  |  | NCD combined | 28.6% |  |
|  |  |  |  |  | Cardiovascular | 13.40% |  |
|  |  |  |  |  | Cancer | 4.4% |  |
|  |  |  |  |  | Respiratory diseases | 1.2% |  |
| WHO (2018) | Ethiopia | | N/A | government report | Mortality due to cardiovascular diseases | 16% | N/A |
|  |  |  |  |  | Mortality due to Cancer | 7% |  |
|  |  |  |  |  | Mortality due to COPD | 2% |  |
|  |  |  |  |  | Mortality due to DM | 2% |  |
| Woldeamanuel, et al. (2019). | Amhara | | 757 | *Data was collected using face to face interview  * The data collection tool was collected based on the American Thoracic Society Division of Lung Diseases questionnaire and British Medical Research Council questionnaire | Respiratory diseases | 11.5% | 10 |
| Wolde et al (2020) | Amhara | | 773 | Interview using the WHO steps survey, physical measurement and sample collection | Diabetes | 6.34% | 10 |
| Wondemagegn, et al. (2017) | Amhara | | 757 | *Data collection tool was developed by reviewing the literature  * For laboratory data, fasting blood glucose was measured as per the WHO recommendations  *Descriptive statistics was done to summarise the characteristics of the study participants | Undiagnosed Diabetes | 11.5% | 10 |
| Worede et al. (2017) | Amhara | | 392 | *Demographic, behavioural, and clinical data of the study participants were collected using a pretested and structured questionnaire  *Blood sample was drawn by a trained medical lab personal | Undiagnosed Diabetes | 2.3% | 10 |
| Yosef (2020) | National (Ethiopia) | | 422 | Face to face data collection using the WHO steps survey and physical measurements such as weight and height was used. | NCD combined | 28.5% | 10 |
| Zekewos et al. (2018) | SNNP | | 2670 | * Data was collected by face to face interview  *General practitioners  conducted physical examination while the nurses collected data on socio-demographic information,  previous history of diabetes and hypertension, and family history of diabetes mellitus and hypertension | Diabetes | 1.9% | 8 |
